# Steric occlusion regulates proximal interactions of acyl carrier protein domain in fungal fatty acid synthase

**DOI:** 10.1038/s42003-020-0997-y

**Published:** 2020-05-29

**Authors:** Jennifer W. Lou, Mohammad T. Mazhab-Jafari

**Affiliations:** 1 Department of Medical Biophysics, University of Toronto, Toronto, Canada; 2 Princess Margaret Cancer Center, University Health Network, Toronto, Ontario Canada

**Keywords:** Cryoelectron microscopy, Enzyme mechanisms, Multienzyme complexes

## Abstract

The acyl carrier protein (ACP) domain shuttles substrates and reaction intermediates in type I fungal fatty acid synthases via transient protein-protein interactions. Here, using electron cryo-microscopy (cryoEM), we report the structure of a fungal FAS stalled at the dehydration reaction, which precedes the final enoyl reduction in the fatty acid biosynthesis cycle. This conformation revealed multiple contact sites between ACP and the dehydratase (DH) and enoyl reductase (ER) domains that occluded the ACP binding to the adjacent ER domain. Our data suggests a minimal path from the DH to the ER reaction site that requires minute changes in the coordinates of the structured N- and C- termini of the ACP domain.

## Abstract (web summary)

Lou and Mazhab-Jafari report the structure of a fungal fatty acid synthase stalled at the dehydration reaction, which precedes the final enoyl reduction in the fatty acid biosynthesis cycle. This study suggests that the binding of acyl carrier protein domain to its proximal dehydratase and enoyl reductase domains in fatty acid synthase is mutually exclusive.

## Introduction

**Fig. 1:**
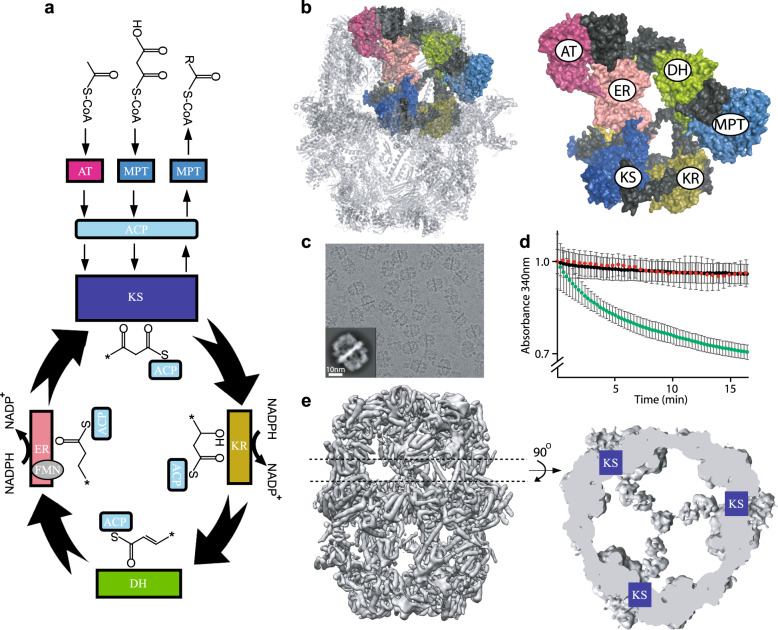
cryoEM analysis of recombinant S. cerevisiae FAS. **a** Schematic of fatty acid synthesis reaction cycle of fungal FAS. **b** Atomic model of *S. cerevisiae* FAS (PDB: 3HMJ) with six catalytic domains highlighted via surface rendering. Color coding same as panel A. **c** Micrograph of recombinant FAS with a 2D class shown as an inset. **d** NADPH consumption assay in the presence (green) and absence (black) of FAS. Red curve is in the presence of 25 μM cerulenin. Error bars are average of three independent protein preparations, each preparation assayed in technical duplicates. **e** cryoEM reconstruction of recombinant FAS (D3 symmetry applied) with a cross section corresponding to ACPs observed at the KS-binding sites.

Lipids constitute one of the major building blocks of life and define the boundaries of cells. They play key roles as signaling molecules and deregulation of lipid metabolism forms the basis of many disorders including type 2 diabetes, cardiovascular diseases, cancer, and infections^1–3^. Therefore, de novo fatty acid synthesis in human and pathogenic species (Fig. 1a) is an attractive drug target. Enzymes responsible for synthesis of the lipid precursor palmitate, a 16-carbon chain hydrocarbon molecule, are collectively known as fatty acid synthases (FAS) and are conserved from bacteria to humans^4^. These enzymes carry out the multi-step synthesis of palmitate from acetyl-coenzyme A (CoA) and malonyl-CoA substrates using NADPH as a reducing agent.

Based on the organization of catalytic domains, two FAS types have been defined. Type II FAS, found in bacteria and plants, is a ‘dissociated system’ where each catalytic enzyme is expressed via a unique gene. Each enzyme works as a monofunctional unit^5^ and transient protein–protein interactions are governed by random diffusion. On the other hand, type I FAS, found in actinobacteria and eukaryotes, is an ‘associated system’ where all essential enzymes are expressed by one or two genes and organized into a large, multi-functional assembly line^6^. Substrate and reaction intermediates are shuttled between catalytic centers via a flexibly tethered acyl carrier protein (ACP) domain.

X-ray crystallographic and cryoEM studies since the late 2000s have advanced our understanding of the quaternary structure of bacterial and fungal type I FAS^7–10^. In fungi, one or two genes encode all the essential catalytic domains and additional segments that serve as structural domains, stabilizing the assembly into a barrel shaped D3 symmetric dodecamer of ~2.6 megadalton in size. The assembly is composed of two C3 symmetric reaction chambers harboring three identical sets of catalytic domains (Fig. 1b) including three ACP domains. The N and C termini of ACP are tethered via long flexible linkers to the wall and central disk of the barrel, respectively. Coarse-grained molecular dynamic simulations^11^ have shown that a single ACP domain may sample the entire space of a reaction chamber.

In the crystal structures of FAS from *Saccharomyces cerevisiae*, sub-nanometer resolution densities corresponding to the ACP are observed only in proximity of the ketoacyl synthase (KS) domain and at a lower signal-to-noise ratio (S/N) compared to other regions of the protein^7,8^. Low resolution (~20 Å) densities with weak S/N are also observed in proximity of other catalytic centers in a cryoEM map of *S. cerevisiae* FAS^12^ that allowed for approximate placement of ACP models in proximity of KS, ketoacyl reductase (KR), ER, and acetyltransferase (AT) domains albeit with ambiguity in ACP orientation. Recent cryoEM studies have observed sub-nanometer resolution ACP densities proximal to KS^13,14^, ER^14,15^, and AT^16^ domains in the apo state of FAS enzymes purified from different fungal species.

The mechanism of ACP-mediated substrate shuttling in fungal FAS is not completely understood. Surface electrostatic complementarity has been suggested as a contributing factor^7–9^. CryoEM studies of endogenous FAS from *S. cerevisiae* and *Candida albicans*^14^ have shown that stalling catalysis at the KS domain using cerulenin, a covalent KS inhibitor, resulted in translocation of ACP toward the inhibited domain in the presence of substrates. Therefore, we asked if it is possible to apply this strategy to other catalytic sites of FAS and image ACP at different locations within the reaction chamber. In this study, we prepared a recombinant FAS from the model organism *S. cerevisiae* allowing mutagenesis for stepwise inactivation of each catalytic site.

## Results

### A model system for investigation of fungal ACP translocation

To enable site-specific inactivation of each catalytic site, we sought to prepare a recombinant FAS to facilitate mutagenesis. Recent studies have shown that the multi-megadalton fungal FAS from *S. cerevisiae* and *Rhodosporidium toruloides* can be purified by heterologous expression in bacteria with the purified complex retaining catalytic activity^17–19^. These observations strongly suggest that the FAS mega-enzymes undergo self assembly. We therefore cloned *FAS1* and *FAS2* genes from *S. cerevisiae* genomic DNA into pET28 and pET15 expression vectors, respectively, and expressed and purified the recombinant FAS from *E. coli* BL21 strain with a 6xHis tag on the C terminus of FAS1 polypeptide (Supplementary Fig. 1a and Supplementary Table 1). CryoEM micrographs of the purified FAS demonstrate fully assembled complex (Fig. 1c and Supplementary Fig. 2) and the protein is functionally active, as judged via consumption of NADPH at 340 nm and is sensitive to cerulenin (Fig. 1d). Importantly, the cryoEM map of the complex shows the global localization of ACP at the KS domain (Fig. 1e and Supplementary Fig. 3) as observed in a previously reported map of endogenous FAS from *S. cerevisiae*^14^. Therefore, this recombinant construct was an ideal model system to study the localization of ACP. All our cryoEM maps reported here are D3 symmetrized (except for localized reconstructions as discussed below) and therefore report on the averaged ACP densities at each catalytic site.

### Effect of stalling catalysis on ACP localization

Using the high-resolution crystal structure of *S. cerevisiae* FAS as a guide^7^, we systematically mutated key residues involved in catalysis to alanine in the AT (S274A), MPT (S1808A), KR (Y839A), DH (H1564A), and ER (H740A) domains (Supplementary Fig. 1a). As expected, these mutations abolished the catalytic activity of FAS (Supplementary Fig. 1b). Since a high-resolution structure of ACP docked at the KS-binding site is available, the KS catalytic residue was not mutated. For the substrate loading/unloading domains, we prepared a FAS that was doubly mutated in both the AT and the MPT domains and added either acetyl-CoA or malonyl-CoA alone to examine the effect on ACP translocation. For the modifying domains (i.e. KR, DH, and ER) we first incubated the purified proteins with excess malonyl-CoA and NADPH to synchronize exposure to the priming substrate (i.e. acetyl-CoA). Excess malonyl-CoA and NADPH were then removed using size exclusion chromatography followed by addition of excess acetyl-CoA, malonyl-CoA, and NADPH. As a control we also compared ACP densities in wild-type FAS in the presence and absence of substrates. Substrates were added to wild-type FAS right before flash freezing the cryoEM grids in liquid ethane to capture the protein under turnover conditions.

**Fig. 2:**
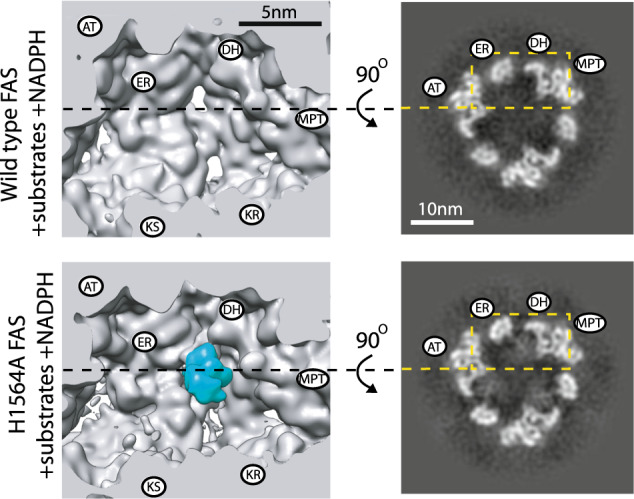
Cross-section of cryoEM density maps of WT and DH (H1564A) mutant FAS in the presence of substrates and NADPH. All maps are D3 symmetric, unsharpened, and low-pass filtered to 7 Å. Slices shown using XIMDISP^32^. The positions of the catalytic sites are highlighted based on the crystal structure of FAS^7^ from one α- and one β-chain with their N and C termini adjacent to each other, respectively. ACP density is highlighted in cyan in the 3D map.

D3 symmetrized cryoEM maps of the WT and mutant proteins were calculated to examine the averaged ACP densities inside the reaction chamber (Supplementary Figs. 2 and 3, and Supplementary Table 2). No noticeable changes in cryoEM densities were observed in the apical region of the reaction chamber near the AT catalytic site (Supplementary Fig. 3). For MPT and KR domains, no changes in ACP density were observed in proximity to the respective inactivated sites (Supplementary Fig. 3). To our surprise, inactivation of FAS at DH in the presence of substrates resulted in strong cryoEM densities in proximity to the catalytic cavities of the DH domains (Fig. 2). Weak densities were observed in the ER-stalled state that partially overlapped with densities observed in the DH-stalled state of the enzyme (Supplementary Fig. 3).

We observed changes in ACP densities docked at the KS (Supplementary Fig. 3), which in this model system is the site with strongest density in the Apo state, suggesting highest occupancy in the absence of substrate. The changes were mostly observed as a weakening of ACP density especially under turnover conditions. It is important to note that the cryoEM density of ACP in proximity of a catalytic site in each 3D reconstruction is dependent on (i) the occupancy of ACP at that site and (ii) the domain dynamics of ACP at the catalytic site. Therefore, the observed changes in ACP densities between WT and mutant FAS are likely a contribution of both aforementioned factors.

### Reconstruction of ACP densities in the DH-stalled FAS

**Fig. 3:**
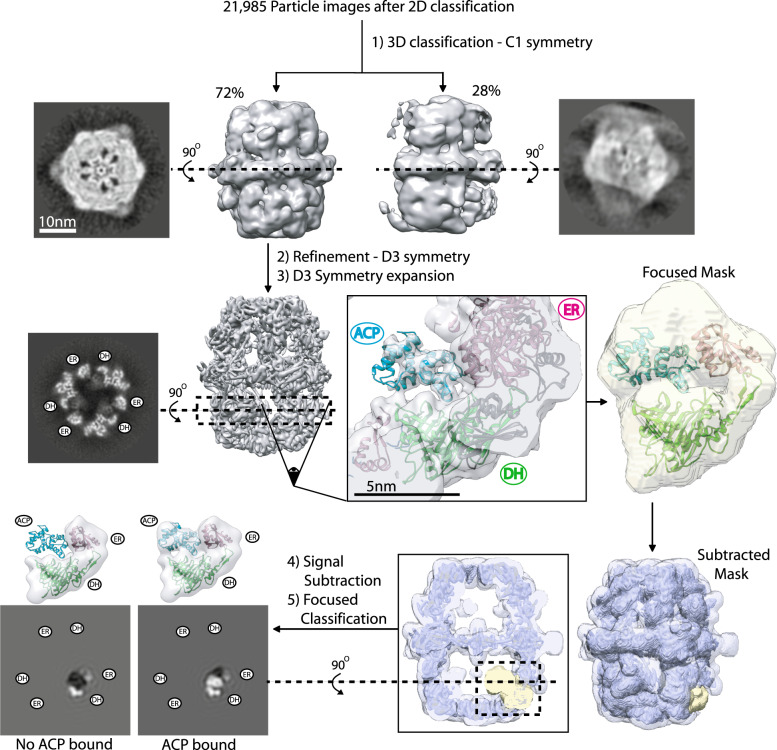
Focused classification of ACP densities observed in the DH-stalled state. Representation of the image analysis procedure. Unsharpened maps are shown. The slices for focused classifications are represented with low-pass filtering to 10 Å.

To the best of our knowledge, fungal ACP has never been seen bound to the DH domain in any type I FAS system. Since we observed strong densities of ACP at the DH domain upon removal of the catalytic histidine inside the cavity of the reaction center, we tested whether it is possible to reliably dock a model of the ACP domain into this density. We collected 329 micrographs of the purified FAS H1564A in the presence of substrates and NADPH (Supplementary Table 3). This dataset gave nearly 16,000 particle images of FAS after 2D and 3D classifications (Fig. 3) to remove contaminants and particles that were partially broken or denatured at the air–water interface^13^. Iterative refinement with D3 symmetry applied resulted in a 4.4 Å resolution map of the mutant FAS with improved density for ACP at the stalled DH site (Fig. 3 and Supplementary Fig. 4a). However, the ACP density was weaker compared to the cryoEM density of the FAS scaffold, suggesting partial occupancy of ACP. Therefore, in the next step we aimed to enrich for DH sites that were occupied by the ACP domain.

Using the globally refined map, we performed a rigid body docking of the ACP model from the crystal structure of FAS^7^. The quality of the ACP density was sufficient to allow confident fitting of six out of eight α-helices of the ACP including all four α-helices of the catalytic lobe (Fig. 3). The resolution was worse for the structural lobe of the shuttling domain (Supplementary Fig. 4a). Using this initial model, we developed a truncated model (Fig. 3) that focused on the observed interface of ACP stalled at the dehydratase reaction.

This truncated model encompassed (i) the docked ACP domain, (ii) two sub-domains of DH, and (iii) part of ER domain that contacted the ACP stalled at the DH domain. This ER fragment also encompassed an ACP binding site that was observed in previous studies of fungal FAS, where the shuttling domain was seen adjacent to the catalytic cavity of the ER domain^14,15^. Our truncated model was ~75 Å in diameter and 50 Å in width and was used to generate a map at 15 Å resolution. A mask encompassing this model and the region of space occupied by ACP bound to the adjacent ER binding site, as previously observed in *C. albicans* FAS, was generated^14^. An overlay of the model with the mask is shown with a transparent yellow surface in Fig. 3. We used focused classification with signal subtraction and symmetry expansion^20^ to improve the quality of the cryoEM map of ACP in the DH-stalled FAS. In our truncated model, all regions corresponding to the FAS scaffold are presumed to be stationary as they have limited conformational variability and have been refined to atomic resolution in previous X-ray and cryoEM reconstructions. The major source of conformational heterogeneity is the location and occupancy of the ACP. A mask of the whole FAS H1564A mutant was generated and the local mask was subtracted from the global mask. The subtracted mask was used to subtract the signal from raw particle images from the consensus refinement, leaving the signal defined by local mask intact. The original particle image dataset was symmetry expanded based on D3 symmetry prior to signal subtraction. The symmetry expanded and signal subtracted dataset was used for focused classification without an orientation search in Relion 3.0. The symmetry expansion produced ~96,000 signal subtracted particle images for 3D classification. We used the cryoEM density of the truncated model at 15 Å as the initial reference for 3D classification, which was limited to five 3D classes. This resulted in one class with strong ACP density for the shuttling domain that represented ~35% of the symmetry-expanded particle images (Fig. 3 and Supplementary Fig. 5). The other classes had lower or no densities corresponding to ACP.

**Fig. 4:**
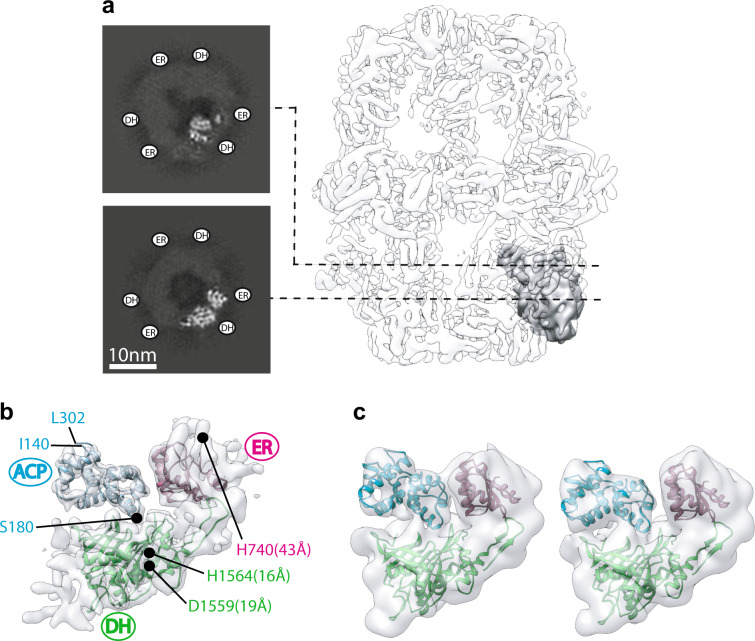
Focused refinement of ACP stalled at the DH domain. **a** Focused region and signal subtracted region are shown in gray and transparent light gray, respectively. Two slices of the unsharpened map are shown to the left. **b** Atomic models (PDB: 2UV8) flexibly fitted to sharpened map. Distances between catalytic residues^5^ of DH (Cδ of D1559 and Nɛ of H1564) and ER (Nɛ of H740) domains to the Cβ of S180 of ACP are given in parenthesis. **c** Focused 3D classification showing domain mobility of ACP stalled at DH. Particle distribution was ~50% for both 3D classes. Both maps are shown at the same threshold and low-pass filtered to 10 Å.

Symmetry-expanded and signal subtracted particle images belonging to the ACP-bound 3D class (Fig. 3) were selected for local refinement in cryoSPARC 2.0 to assess the global and local resolution of the focused map. The local refinement was initiated with a 20-Å low-pass filtered map of the original truncated model and the focused mask used in Relion 3.0. Local refinement demonstrated a global resolution of 5.8 Å (Fig. 4a, b and Supplementary Fig. 6). The region of the map corresponding to ER and DH densities were estimated to have a resolution between 5 and 6 Å and a resolution range of 5 to 9 Å was estimated from the catalytic lobe to the structural lobe of ACP (Supplementary Fig. 6c). Fitting of the atomic model of the ACP domain into the local refined map (Fig. 4b and Supplementary Fig. 6d) demonstrated corresponding densities for all helices of the shuttling domain and placed serine 180 (i.e. the phosphopantetheine attachment site) in proximity of the catalytic cavity of the DH reaction site in a similar conformation to that seen^21^ for bacterial type II FAS (Supplementary Fig. 7a, b). The hydroxyl group of the nascent fatty acid chain resides ~4 Å away from the covalently attached phosphopantetheine prosthetic group, which in turn can extend to ~18 Å^22^. Therefore, the observed ACP position is in range for catalysis by the DH domain. The N and C termini of ACP face away from the reaction cavity of the DH domain. This model indicates that both the structural and the canonical lobes of ACP form transient contacts with the chamber scaffold with primarily polar and charged residues lining the interface (Supplementary Fig. 8). Further focused 3D classification in Relion 3.0 on this occupied class demonstrated the conformational heterogeneity of ACP docked at the stalled DH site representing a wobbling motion of the ACP between the ER and the DH domains (Fig. 4c). This displacement explains, at least in part, the local resolution variation observed for the stalled ACP (Supplementary Fig. 6c).

### Reconstruction of ACP densities in the ER-stalled FAS

**Fig. 5:**
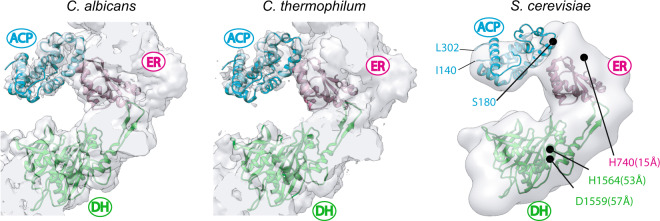
ACP docked at the ER domain. Comparison of cryoEM densities of ACP observed at the ER domain of endogenous FAS from *C. albicans* and *C. thermophilum* with that of the ER-stalled ACP in recombinant *S. cerevisiae* FAS. ACP coordinates in the *S. cerevisiae* map (right) and distance measurements are based on *C. albicans* model PDB: 6U5V^14^. Distances are as described in Fig. 4b.

Following the dehydration reaction, an enoyl reduction is catalyzed by the ER domain. Since we were able to observe weak densities in the vicinity of the ER domain in the ER-stalled FAS (Supplementary Fig. 3), we sought to carry out similar focused classification to purify in silico the ER sites that were occupied by ACP. We collected 258 micrographs of ER-stalled FAS. The number of particles per micrograph was higher for this dataset compared to our DH-stalled sample and we were able to select ~23,000 particle images of ER-stalled FAS after 2D and 3D classification to remove contaminants and broken particles (Supplementary Fig. 9a and Supplementary Table 3). Iterative refinement of selected particles with D3 symmetry applied generated a map with a global resolution of 4.2 Å (Supplementary Fig. 4b). Weak densities were observed inside the reaction chamber of ER-stalled FAS in proximity to the ER binding sites. Using a method similar to that described in the previous section, we generated a symmetry expanded and signal subtracted particle stack with ~135,000 unique ER sites for the ER-stalled state (Supplementary Fig. 9a). We used the same mask that encompassed the same region of the FAS scaffold as described in the previous section for the DH-stalled FAS. The initial reference map for the focused 3D classification was modified to include ACP docked at the ER as previously observed in apo FAS from other species^14,15^ and low-pass filtered to 15 Å. Using this map and mask, we carried out a series of focused 3D classifications without an orientation search (Supplementary Fig. 9b). After three rounds of classification, 3D classes with ACP density (Supplementary Fig. 9c) at the ER domain were generated with binding similar to that observed for two other fungal FAS systems (Fig. 5). The final 3D classes containing the ACP signal represented ~10% of the unique ER sites in the dataset (i.e. 13,500 signal subtracted and symmetry expanded particle images).

## Discussion

A pre-requisite for substrate-shuttling (i.e. shuttling of acyl-modified ACP) is weak and transient interactions between the ACP and the interior of the FAS reaction chamber. The localization of the ACP domain that we observed upon stalling catalysis at the dehydratase step provided the opportunity to define the binding site of this shuttling domain at the DH. Both the catalytic and structural lobes of the ACP interact (see review in Maier et al.^6^) with multiple elements in the β-chain. The structural domain of the ACP has so far only been observed to contact the structural insertion of the KS domain that forms part of the central α-disc of the FAS barrel^7,8^. The interaction of ACP with the ER^14,15^ and AT^16^ domains is shown to be mediated by its catalytic lobe, with no contacts between the structural lobe of the shuttling domain and the β-chains. The mode of interaction of ACP with ER is likely conserved between different species of fungi, as similar relative orientations have been observed in at least three different species (Fig. 5) and the residues that form the ACP binding site on the ER surface are highly conserved (Supplementary Fig. 8). The interaction interface of ACP with the DH domain covers a larger surface area and involves multiple structural elements contributed by both the DH and the ER domains. The residues that line this multi-faceted interface are not conserved between fungal species (Supplementary Fig. 8). However, examining the frequency of amino acids in fungal species lining the interface for ACP interaction, reveals a general preservation of chemical and geometric amino acid characteristics (Supplementary Fig. 8). It is likely that weak electrostatic contacts are sufficient for transient association of ACP for catalysis at the DH domain.

**Fig. 6:**
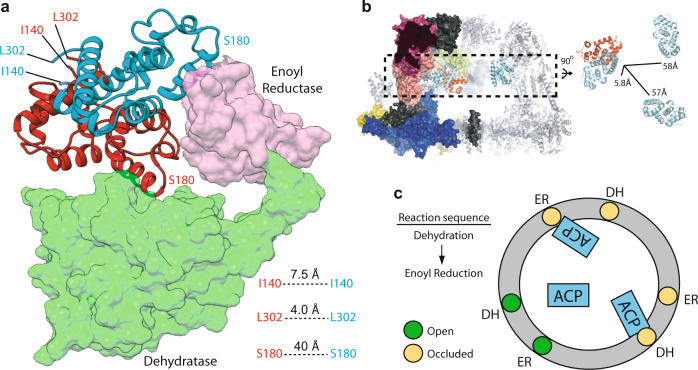
Steric occlusion of ACP at the proximal DH and ER domains. **a** Overlay of ACP docked at the proximal DH (cartoon, red) and ER (cartoon, cyan) domains. Distances are between Cα atoms. **b** Averaged distances from the N- and C-termini of ACP docked at the DH to the three possible ACP binding sites at the ER domains (PDB: 6U5V), within one reaction chamber (PDB: 2UV8). **c** A simplified 2D representation for the mutually exclusive binding mode of ACP to proximal DH and ER domains.

The location of the ACP at the stalled DH catalytic site overlaps with that of ACP at the adjacent ER (Fig. 6a). There are steric clashes between both structural and catalytic lobes of ACP docked at the DH domain with the structural lobe of ACP docked at the ER domain. Therefore, when one of the DH sites is occupied by an ACP domain the adjacent ER of the same β-chain protomer is blocked for optimal ACP binding by the other two shuttling domains within the reaction chamber (Fig. 6b, c). Similarly, if one ACP binds the ER domain, the adjacent DH domain of the same β-chain protomer will be blocked for optimal binding of the other two ACPs. This mode of binding mediates mutually exclusive access to the shuttling domains for binding to domains catalyzing the final two steps of the fatty acid synthesis cycle. Based on our model, minimal changes in the locations of the structured N and C termini of the ACP (i.e. I140 and L302 based on solution NMR structures of isolated *S. cerevisiae* ACP^23^) can satisfy a domain translocation of the ACP from the DH domain to the proximal ER. Therefore, no changes in the ACP N- and C-terminal linker conformation are needed for transition between these successive reactions. This contrasts with an order of magnitude larger displacement of ACP linkers if an ACP docked at a DH domain were to translocate to an ER domain of another β-chain protomer (Fig. 6b). This mode of binding between the DH and ER domains of the same β-chain could be highly advantageous for the reaction sequence of fatty acid synthesis, as (i) docking of an ACP at one DH site will occlude optimal binding of other ACPs to the adjacent ER site and (ii) a DH to ER transition may be satisfied mainly by a domain rotation with a pivot centered at the structural lobe of the ACP. In other words, upon catalysis by DH, the next step of the reaction (i.e. ER of the same β-chain) is ‘reserved for optimal ACP binding conformation’ via occlusion of the other two ACPs and may be accessed with minor changes in the ACP flexible linkers. Previous MD simulations of the ACP interaction landscape in the absence of substrates has shown that movements of the shuttling domain are stochastic^11^ with higher probability of a ACP domain interacting with proximal DH and ER domains. It is interesting to note that residues in the DH and ER domains that line the observed ACP interfaces in the DH- and ER-stalled states form a continuous support layer for interaction with the shuttling domain (Supplementary Fig. 8).

## Methods

### Cloning

Genomic DNA from the haploid protease deficient *S. cerevisiae* BJ2168 plasmid (MATa leu2 trp1 ura3–52 prb1-1122 pep4-3 prc1-407 gal2) was obtained by phenol/chloroform extraction. *FAS1* and *FAS2* genes were amplified from genomic DNA using primers containing overlaps with the vector backbone for cloning with NEBuilder HiFi DNA Assembly (NEB) (Supplementary Table 1). *FAS1* was cloned into NcoI-digested pET-28a(+) with a C-terminal His_6_ tag (JWL02). *FAS2* was cloned into NcoI-digested pET-15b(+) (JWL03). The sequences of the *FAS1* and *FAS2* genes were verified (Supplemantary Data 1) via whole plasmid sequencing of JWL02 and JWL03 at the Center for Computational & Integrative Biology DNA core facility in Massachusetts General Hospital.

Point mutations were generated by the overlap extension method^24^ using a forward primer containing the mutation and a reverse primer 200–2000 bp downstream (Supplementary Table 1). PCR fragments were purified using spin columns and used as megaprimers to amplify the entire plasmid and introduce the point mutation. Mutations were verified by Sanger sequencing using custom sequencing primers (Supplementary Table 1).

### Transformation, expression, and protein purification

The JWL02 and JWL03 plasmids were co-transformed into *Escherichia coli* BL21 cells by electroporation, plated on LB agar plates containing 50 μg/mL kanamycin and 100 μg/mL ampicillin, and grown overnight at 37 °C. One colony was inoculated into 50 mL LB media containing kanamycin and ampicillin and grown overnight at 37 °C, 250 RPM. In all, 10 mL of this starter culture was transferred to 1 L LB media containing kanamycin and ampicillin in a 4-L flask and grown at 37 °C, 180 RPM in an Innova 42 shaker (New Brunswick) until the OD_600 nm_ reached 0.6. The culture was cooled to 15 °C and expression was induced with 0.5 mM IPTG. Expression occurred at 15 °C, 180 RPM overnight.

Cells were harvested by centrifugation at 4000 × *g* for 15 min and resuspended in lysis buffer (200 mM potassium phosphate pH 7.4, 300 mM KCl, 10 mM imidazole, 5 mM β-mercaptoethanol, 0.5 mM PMSF, 1 mM benzamidine, and 5 mM aminocaproic acid). Resuspended cells were incubated with DNAse I and lysozyme for 10 min. Cells were lysed by sonication (Branson Analog Sonifier S-450A) in an ice-water bath with five cycles of ~15 W pulses every 0.5 s for 1 min followed by 2 min rest. The lysate was cleared by centrifugation at 40,000 × *g* for 1 h. The cleared lysate was filtered using a 0.22-μm filter and loaded onto a pre-equilibrated 5 mL HisTrap column (GE Healthcare). The column was washed with 10 column volumes of wash buffer (200 mM potassium phosphate pH 7.4, 300 mM KCl, 20 mM imidazole, and 5 mM β-mercaptoethanol) before elution with a linear gradient of 0–100% elution buffer (200 mM potassium phosphate pH 7.4, 150 mM KCl, 300 mM imidazole, 5 mM β-mercaptoethanol) over 20 column volumes. Fractions were pooled and concentrated to 1 mL and injected into a Superose6 10/300 GL column (GE Healthcare) pre-equilibrated with TBS (50 mM Tris pH 7.4, 150 mM NaCl). Gel filtration fractions were pooled and concentrated to 2 mg/mL, flash frozen in liquid nitrogen and stored at −80 °C. For cryoEM samples, proteins were used fresh on the day of purification.

### Activity assays

Activity assays of purified recombinant FAS were performed as described^14^. Briefly, 20 μg FAS was mixed with 0.2 mM acetyl-CoA, 0.7 mM NADPH, 1 mM DTT, and 100 mM potassium phosphate pH 7.4 in a 100-μL reaction. The level of NADPH was monitored at 340 nm for 3 min and the reaction was started by adding 30 nM malonyl-CoA and monitored for 1 h. The specific activity of wild type recombinant FAS was measured from three independent protein preparations to be 78 ± 32 mU/mg, where one unit is defined as consumption of 1 μmol of malonyl-CoA per minute. Only linear part of the curve (i.e. 2 min post malonyl-CoA addition) was used for calculation of the specific activity^25^.

### cryoEM sample preparation and image collection

To prepare samples for CryoEM imaging, 3 μl of freshly purified protein complexes were added at a concentration of 1–2 mg/ml to glow-discharged (25 s in air) holey gold grids^26^ mounted in a Vitrobot Mark IV. Blotting was done for 3 s at 4 °C and 100% humidity before plunge freezing in liquid ethane. A total of three grids were prepared per condition reported in Supplementary Table 2 and Supplementary Table 3 from the same purifications. Data reported in Supplementary Table 2 were collected from one grid and data reported in Supplementary Table 3 were collected from three grids.

Electron micrographs were collected with a FEI Tecnai F20 field emission electron microscope equipped with a Gatan K2 summit direct detector device (DDD) camera. Micrographs were acquired as movies in counting mode using DigitalMicrograph® software with 1.45 Å/pixel, 2 frames/s for 15 s, and an exposure rate of 1.2 e^−^/Å^2^/frame (Supplementary Table 2 and Supplemantary Table 3). Images were converted to mrc format using dm2mrc^27^.

Stock solutions of acetyl-CoA, malonyl-CoA, and NADPH were prepared at a concentration of 10 mM in TBS buffer. For the AT and MPT double mutant FAS construct (S274A - S1808A), 1 μL of either acetyl-CoA or malonyl-CoA stock solutions were diluted with TBS to 5 μL before addition to 10 μg (at 2 mg/ml) of purified FAS, before application onto cryoEM grids.

For KR (Y839A), DH (H1564A), and ER (H740A) mutants, 100 nmol NADPH and 50 nmol malonyl-CoA were added to the pooled and concentrated HisTrap fractions (final volume 0.5 ml) and incubated for 1 h at room temperature. Substrates were removed by gel filtration as described above. In total, 10 μg (at 2 mg/ml) of purified FAS was mixed with a 5-μL solution containing 10 nmol acetyl-CoA, 25 nmol NADPH, and 15 nmol malonyl-CoA. Samples were kept on ice for 30 min before application onto cryo-EM grids.

For the WT FAS, 10 μg of purified FAS at 2 mg/ml was mixed with solutions containing either 5 μL of TBS (for the apo sample), or 5 μL of TBS containing 10 nmol acetyl-CoA, 25 nmol NADPH, and 15 nmol malonyl-CoA (for turn-over conditions) on ice, respectively. Samples were applied onto the cryoEM grids immediately after the addition of TBS plus substrates.

### Image processing

Data presented in Supplementary Table 2 were processed with cryoSPARC 2.0^28^. Movies were aligned by *alignframe_lmbfgs* implemented in cryoSPARC 2.0^29^. CTF estimation was done with CTFFIND4^30^. Particle motion correction was performed with *alignpart_lmbfgs* implemented in cryoSPARC 2.0^29^. High resolution refinement was done with the homogenous refinement algorithm using particles selected from reference free 2D classification. A 30-Å low-pass filtered cryoEM map was generated from PDB: 2UV8^7^ with ACP atoms deleted from the model. This map was used as the initial reference in the homogenous refinement. Model to map conversion was carried out in *UCSF* Chimera^31^. Low-pass filtering of refined maps was carried out using the ‘relion_image_handler’ command. All one-voxel-thin slices were generated using XIMDISP^32^.

Data presented in Supplementary Table 3 were processed in Relion 3.0^33^. Movies were aligned with the Relion implementation of MotionCor2 and CTF estimation was performed using CTFFIND4. Following 2D classification, all 3D classification was done with C1 symmetry using a 30-Å low-pass filtered cryoEM map generated from PDB 2UV8 with ACP atoms deleted as the initial reference. Particle images were selected from 3D classes and refinement was done with C1 symmetry. The refined maps were aligned to the D3 symmetry axis and a second refinement was carried out with D3 symmetry imposed. The reference was aligned to the symmetry axis using the ‘relion_align_symmetry’ command.

### Focused classification

A mask for focused classification was made by rigid body docking of a model of a β-chain protomer and the ACP domain from an α-chain protomer from the FAS crystal structure (PDB: 2UV8) to an asymmetric unit of the D3 refined map of the DH-stalled FAS. Residues from the ER and DH domains of the β-chain were selected based on proximity to the observed ACP density in the DH-stalled state and the domain boundaries identified in a previous crystallographic study of *S. cerevisiae* FAS^7,8^. The residues were 601–704 (ER domain, encompassing the ACP binding sites observed in *C. albicans* and *T. thermophilum*) and residues 1236–1658 (DH pseudo-dimer). ACP residues 140–302 from the α-chain of FAS (PDB: 2UV8) were rigid body docked into the D3 refined map of DH-stalled FAS in the asymmetric unit with the docked β-chain fragments. This model comprising partial segments of ER and DH domains plus the whole ACP domain docked at the observed ACP density in the DH-stalled state was used to generate the initial reference and was low-pass filtered to 15 Å for the focused classifications described below. To create a mask that covered the partial model and the position of the ACP in the ER binding mode, we expanded the aforementioned partial model of ER-DH -ACP^DH^ by including a second model of the ACP domain docked at the ER binding site based on homology to previously observed ACP bound to ER in fungal FAS (PDB: 6U5V)^14^. A 15-Å resolution mask (i.e. focused mask) comprising the residues mentioned above was generated with UCSF chimera and Relion 3.0. The focused mask was extended by 3 pixels (1.45 Å/pixel) with 3 pixel padding. This mask was subtracted from a mask generated from the D3 refined map of DH-stalled FAS to produce a subtracted mask. The subtracted mask was then multiplied by the map and the ‘modified map’ containing all regions of FAS except for the region defined by the focused mask, was used for subsequent steps.

The rotation matrix for each asymmetric unit of FAS (i.e. α-,β-chain heterodimer) was computed using the original particle images with alignment information from the D3 symmetry refinement (done in Relion 3.0) via the ‘relion_particle_symmetry_expand’ command^20,34^. Different projections from the ‘modified map’ were then generated based on orientation information from the D3 symmetry refinement. These projections were subtracted from the symmetry expanded particle images. Subtracted images were used for focused 3D classification without orientation search in Relion 3.0 with an optimized regularization parameter of 500. The focused refinement was done in cryoSPARC 2.0 by importing the subtracted particle data set from Relion 3.0 belonging to the ACP containing 3D class in Fig. 3 (i.e. Class 1 in Supplementary Fig. 5) low-pass filtered to 20 Å as the initial reference. The mask for the cryoSPARC focused refinement was generated using the initial reference map in Relion 3.0 and low-pass filtered to 15 Å and expanded by 3 pixels with 3 pixels padding. A B factor of 344 was estimated using Guinier plot analysis implemented in cryoSPARC and used for map sharpening. A local resolution estimate of the focused refined map was done using cryoSPARC 2.0.

### Model fitting and visualization

Model fitting was done by rigid body docking of the selected regions of ER, DH, and ACP domains, as described in the manuscript text, into the cryoEM maps of the stalled complexes. An atomic model of FAS (PDB: 2UV8^7^) was used for docking. Maps and models were visualized with UCSF chimera^31^ and PyMol^35^. Flexible fitting was done for Fig. 4b and Supplementary Fig. 6d using PHENIX real space refinement with the resolution limited to 6 Å. The Ramachandran statistics were: 83.8% favored, 15.85% allowed, and 0.35% outlier. For the distance measurements reported in Figs. 4b and 5, side chain conformations for residues H740, D1559, and H1564 were from PDB: 2UV8.

### Sequence alignment

Non-redundant protein sequences corresponding to the subphylum *Saccharomycotina* (i.e. true yeast, taxonomy id: 147537) were chosen for alignment using BLASTp^36^. The β-chain sequence of *S. cerevisiae* FAS was chosen as the search template. A maximum number of 100 sequences was chosen for initial display. Sequences belonging to different strains of *S. cerevisiae* were excluded from the analysis. The remainder encompassed 58 sequences from other species of *Saccharomycotina* (Supplementary Data 2). Multiple sequence alignment was done with Clustal Omega^37^. LOGO plots were generated via the WebLogo server^38^ with the y-axis set to represent frequency of amino acids plotted against amino acid number (based on *S. cerevisiae* sequence) on the x-axis.

### Reporting summary

Further information on research design is available in the Nature Research Reporting Summary linked to this article.

## Supplementary information

Supplementary Information

Description of Additional Supplementary Files

Supplementary Data 1

Supplementary Data 2

Supplementary Data 3

Reporting Summary

## Data Availability

Map and Model of DH-stalled FAS are available with PDB code 6WC7. All relevant data are available from the corresponding author upon request. Whole-plasmid sequencing results for FAS1 and FAS2 genes cloned from S. cerevisiae genomic DNA are shown in Supplementary Data 1. Sequences of fungal species used in multi-sequence alignment are shown in Supplementary Data 2. The source data underlying Fig. 1d are provided in Supplementary Data 3.
