# Supplementary material for: Steric occlusion regulates proximal interactions of acyl carrier protein domain in fungal fatty acid synthase
Source: Commun Biol. 2020 May 29;3:274. doi: 10.1038/s42003-020-0997-y (PMC7260205; doi:10.1038/s42003-020-0997-y)
Supplement: Supplementary file 1 — Supplementary Information [file 42003_2020_997_MOESM1_ESM.pdf]

Supplementary Information for

Steric occlusion regulates proximal interactions of acyl carrier protein domain in fungal fatty acid synthase

Jennifer W. Lou[1], Mohammad T. Mazhab-Jafari[1,2*].
[1]Department of Medical Biophysics, University of Toronto, [2]Princess Margaret Cancer Center, University Health Network, Toronto, Ontario, Canada.

* Correspondence: Mohammad.Mazhab-Jafari@uhnresearch.ca

This PDF file includes:

Supplementary Figure 1……………………………………………..2
Supplementary Figure 2…………………………..…...………..3-4
Supplementary Figure 3……………………………….…………..5
Supplementary Figure 4……………………………….…………..6
Supplementary Figure 5……………………………….…………..7
Supplementary Figure 6……………………..………...………..8
Supplementary Figure 7…………………………….……………..9
Supplementary Figure 8…………………………….…………….10
Supplementary Figure 9…………………………….…………….11-12
Supplementary Table 1…………………………...……..……13
Supplementary Table 2…………………………………………14
Supplementary Table 3……………………………...……………15
Supplementary References………………………………………..16

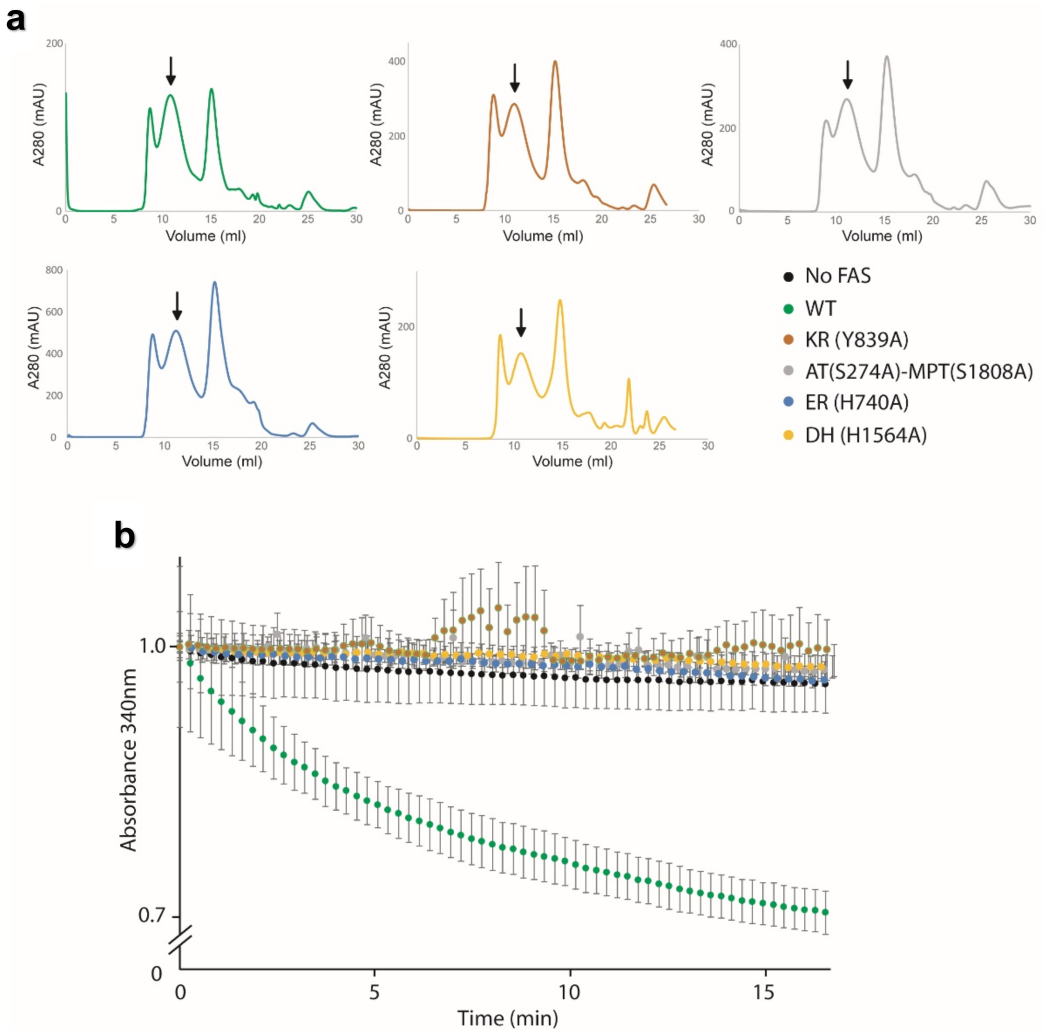

Supplementary Figure 1. Purification and functional assay of recombinant FAS WT and mutants. a) size exclusion chromatographs (Superose 6 increase) of WT and mutant FAS complexes expressed in *E. coli*. Peaks corresponding to fully assembled FAS are shown with arrows. **b)** functional assay of purified FAS. Color coding same as in panel A. WT FAS activity assay is performed with three independent protein preparations, each with a technical duplicate. For mutant FAS, the error bars represent data from one protein preparation per mutant, with a technical triplicate.

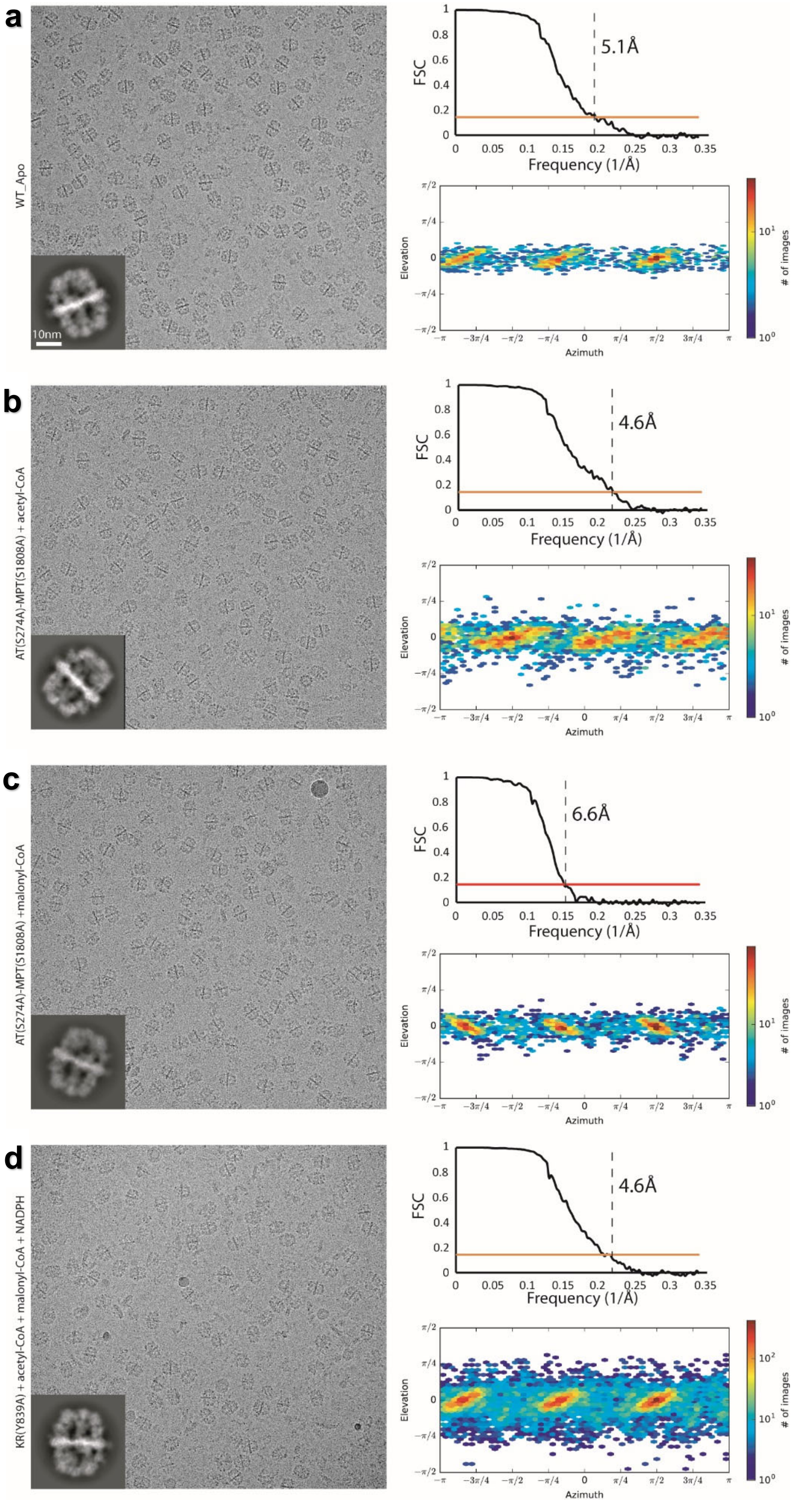
a
WT_Apo
10nm
FSC
5.1Å
Frequency (1/Å)
Elevation
Azimuth
# of images
b
AT(S274A)-MPT(S1808A) + acetyl-CoA
FSC
4.6Å
Frequency (1/Å)
Elevation
Azimuth
# of images
c
AT(S274A)-MPT(S1808A) +malonyl-CoA
FSC
6.6Å
Frequency (1/Å)
Elevation
Azimuth
# of images
d
KR(Y839A) + acetyl-CoA + malonyl-CoA + NADPH
FSC
4.6Å
Frequency (1/Å)
Elevation
Azimuth
# of images

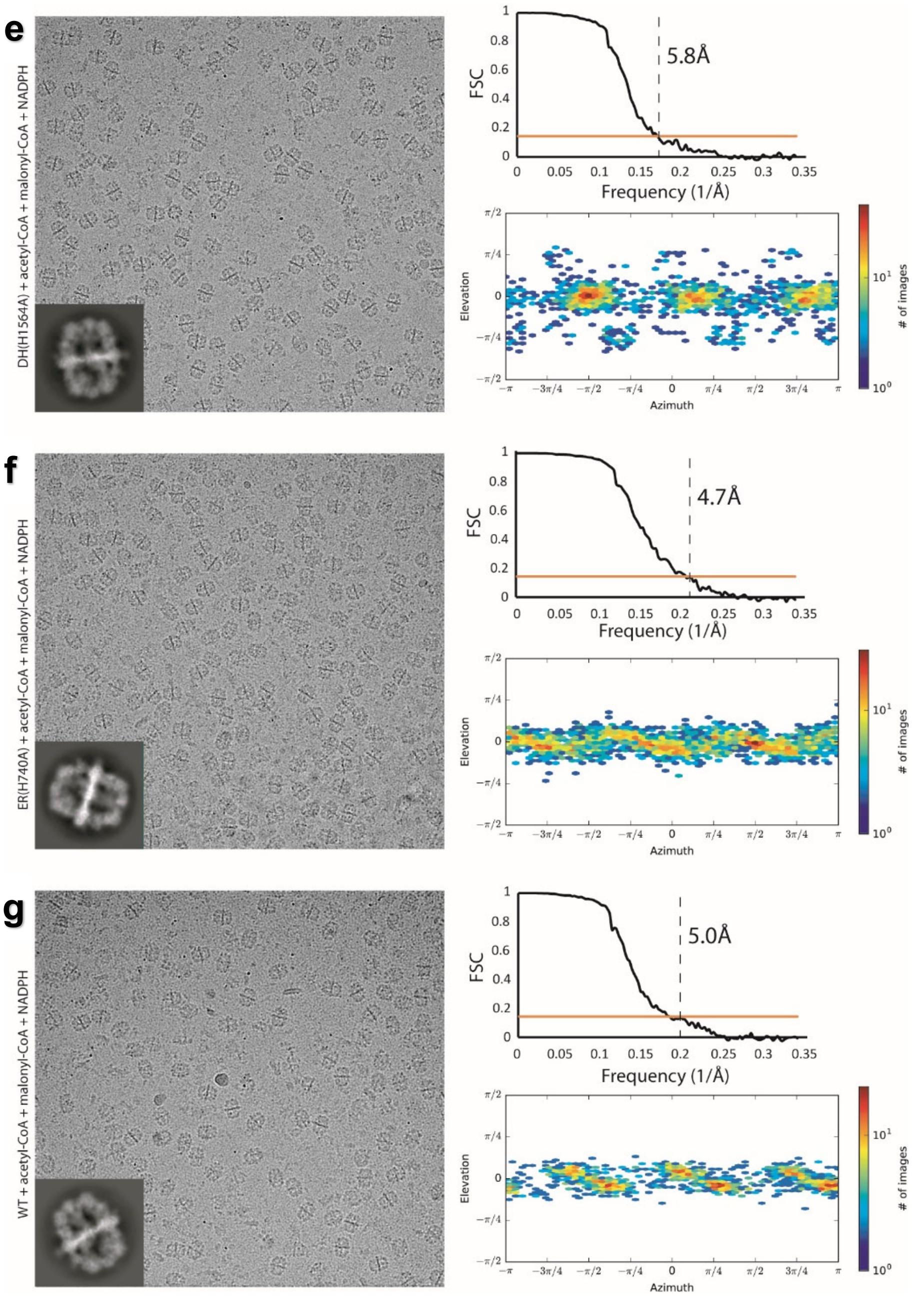

Supplementary Figure 2. cryoEM analysis of purified FAS. Representative electron micrographs for recombinant FAS WT and mutants in vitreous ice. Sample conditions are indicated to the left of each micrograph in **a-g**. To the right of each panel: mask corrected FSC curves (top, right) and orientation distributions of particle images (bottom, right). See table S2 for data collection and image processing details.

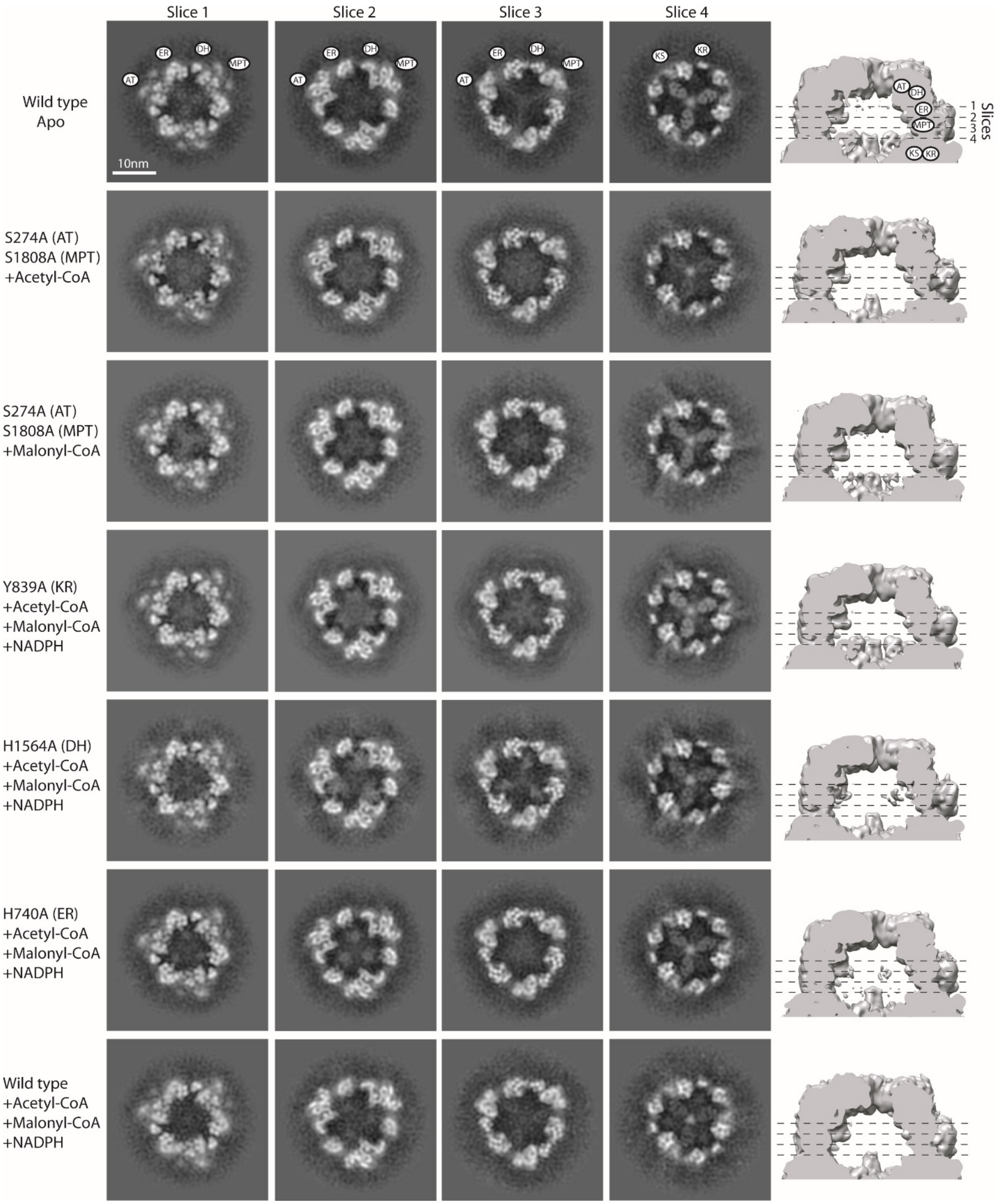

Supplementary Figure 3. Cross-sections of cryoEM density maps reported in supplementary table 2. All maps are D3 symmetric and low pass filtered to 7 Å for comparison. Slices shown using XIMDISP[1]. One chamber of each map is shown at the end of each row with the location of slices highlighted in the top row. Locations of catalytic centers are shown in the first row based on crystal structure of FAS (PDB: 2UV8[2]) as described in figure 2.

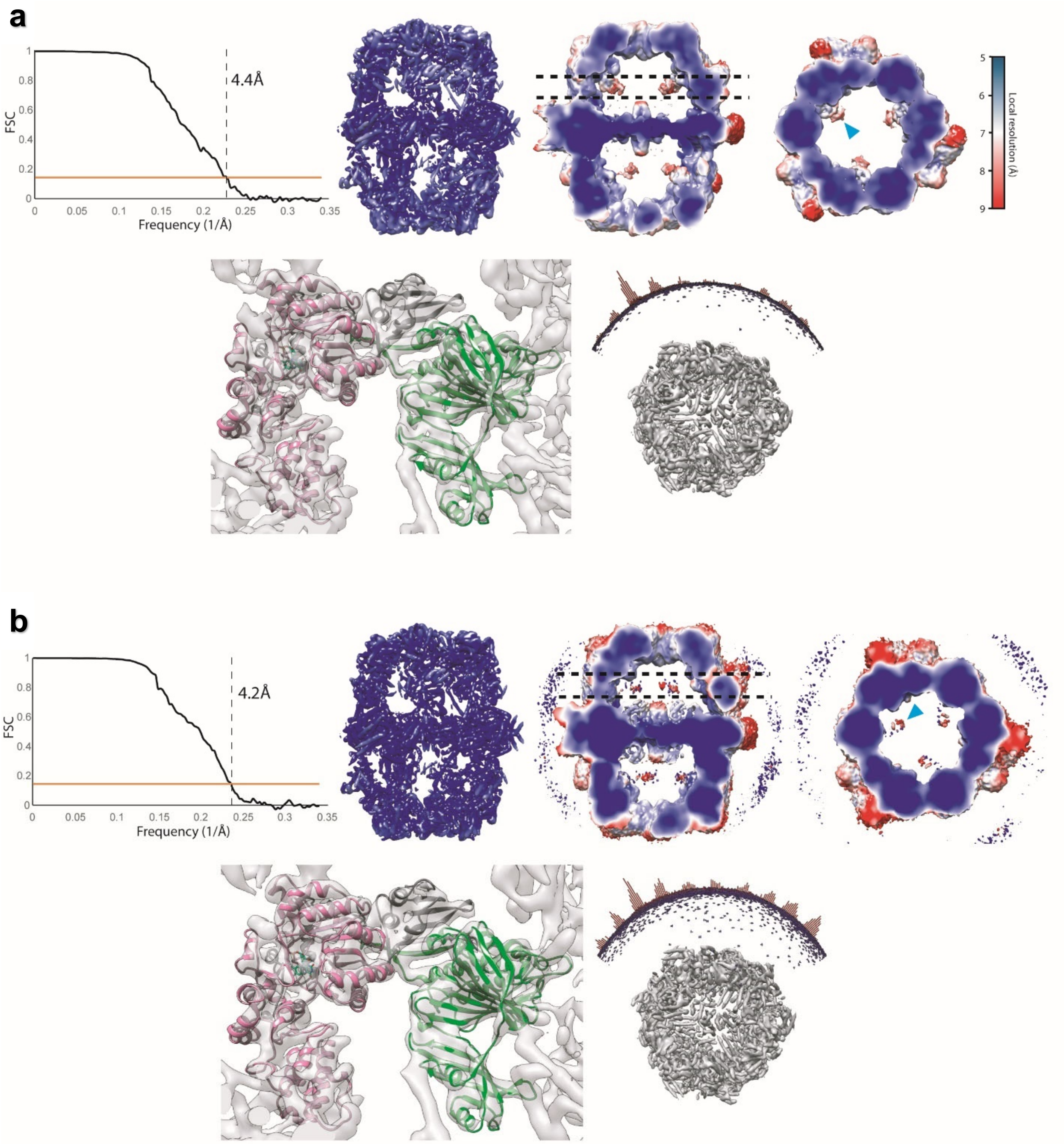

Supplementary Figure 4. Quality of the cryoEM maps of DH and ER stalled states used in the focused classification and reported in table S3. Mask corrected FSC of the cryoEM map, local resolution estimate, particle orientation distribution, and example of model to map fit for **a)** DH- and **b)** ER-stalled datasets used in focused classification with Relion 3.0. Blue arrows indicate density corresponding to ACP domain.

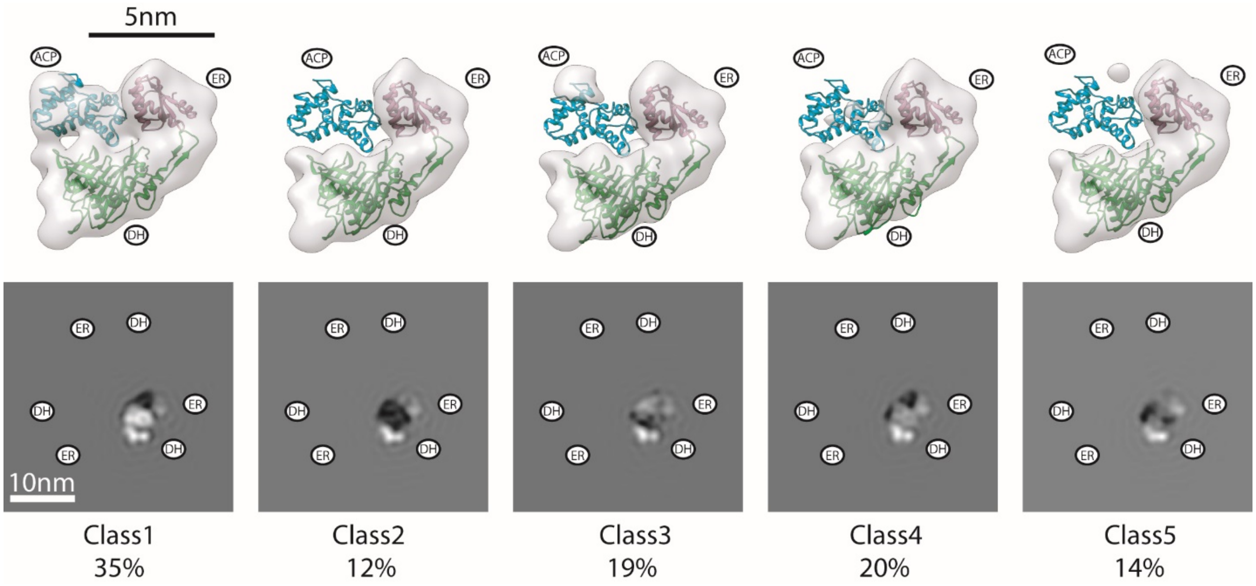

Supplementary Figure 5. Focused 3D classes for the DH-stalled FAS. cryoEM density map of each class is shown on the top with the slice though each of the classes shown at the bottom. The position of the slice is shown in figure 3. The percentage number of particles for each class is indicated bellow each slice. The maps are shown at identical threshold and maps and cross sections are low pass filtered to 10 Å for comparison.

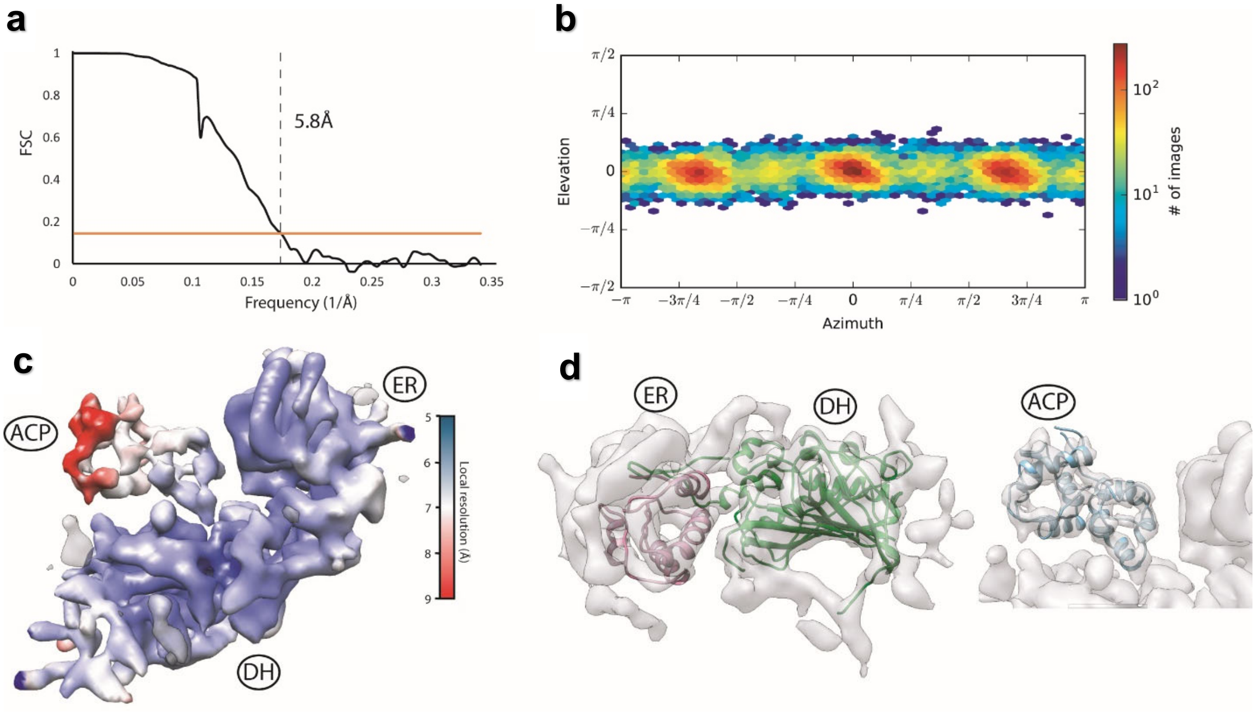

Supplementary Figure 6. Quality of the focused refined map of DH stalled recombinant FAS. a) Mask corrected FSC for the focused refinement of symmetry expanded and signal subtracted particle images for the DH-stalled state of FAS. **b)** Particle orientation distribution. **c)** local resolution estimate. **d)** Model to map fit for ACP and ER-DH fragments of the model.

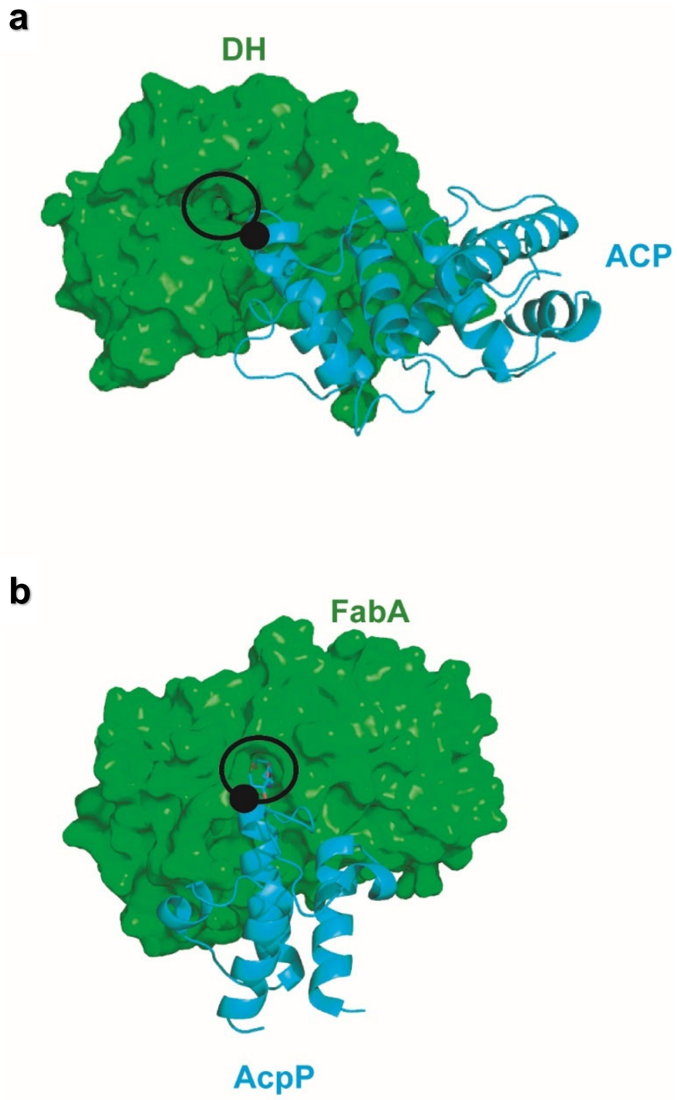

Supplementary Figure 7. Fungal type I and bacterial type II FAS comparison for ACP interaction with dehydratase. Comparison of **a)** ACP model in DH-stalled fungal type I FAS to **b)** AcpP cross-linked to the catalytic histidine of FabA in bacterial type II FAS (PDB: 4KEH[3]). Black circles and dots represent positions of the catalytic cavity of DH and the serine residue that is modified in cells by attachment of the phosphopantetheine arm, respectively.

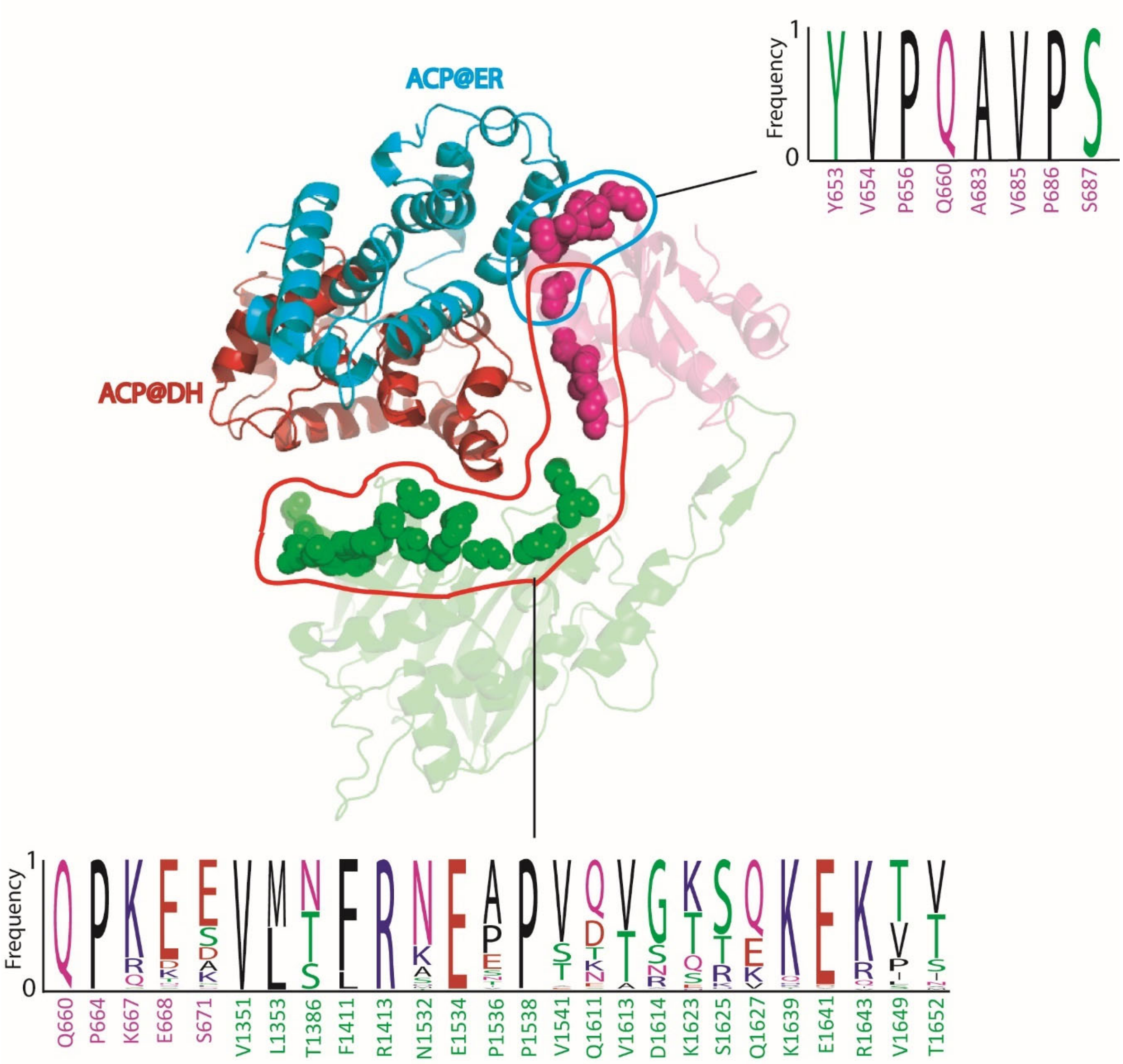

Supplementary Figure 8. Conservation of residues lining the interface of ACP binding sites on ER and DH domains. Model PDB: 2UV8. Backbone atoms of residues with solvent exposed sidechains are shown with spheres. ER and DH domains are shown as transparent cartoons with same color coding as figure 1. Residues belonging to each domain is colored respectively. ACP docked at ER and DH are shown with non-transparent cyan, and red cartoons respectively. Sequence alignment represented with Logo plot. Cyan and red enclosed solid lines highlights residues facing ACP docked at ER and DH domains, respectively.

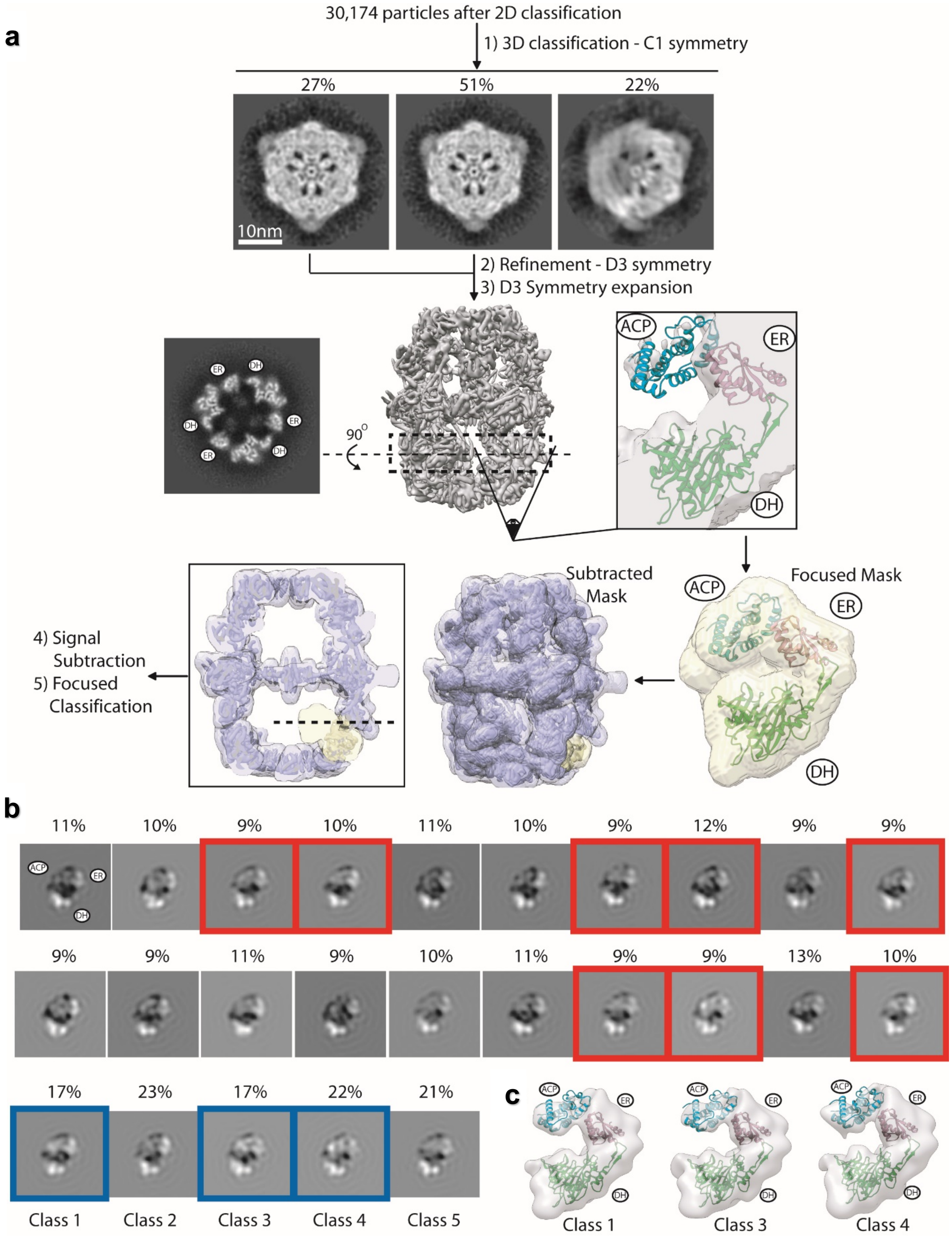
a
30,174 particles after 2D classification
1) 3D classification - C1 symmetry
27%
51%
22%
10nm
2) Refinement - D3 symmetry
3) D3 Symmetry expansion
90°
ACP
ER
DH
Subtracted Mask
Focused Mask
4) Signal Subtraction
5) Focused Classification
b
11%
10%
9%
10%
11%
10%
9%
12%
9%
9%
9%
9%
11%
9%
10%
11%
9%
9%
13%
10%
17%
23%
17%
22%
21%
Class 1
Class 2
Class 3
Class 4
Class 5
c
Class 1
Class 3
Class 4

Supplementary Figure 9. Focused classification of ACP in the ER stalled state of recombinant FAS. a) Visual representation of the procedure is shown. **b)** Cross section of 3D classes shown (dashed line in lower left panel of A). Highlighted in red are classes chosen for the next round of 3D classification (represented in each row). Particle distribution are indicated above each 3D class as a percentage of total particles after 3D classification in panel A. The classes containing the ACP signal are highlighted in blue in the final round of 3D classification and **c)** shown as 3D cryoEM maps. Maps and cross sections in B) and C) are low passed filtered to 10 Å.

Supplementary Table 1. Primers used in this study. Primers used for cloning of FAS genes into bacterial expression vectors are with 'NEB' prefix. Primers for mutagenesis are with FAS domain abbreviation prefix. Custom primers used for sequencing are indicated with suffix 'seq'.

Primer name	Sequence (5' -> 3')
NEB_fas2_F	tgtgatgatgatgatgatggctgctgccCTATTTCTTAGTAGAAACGGC
NEB_fas2_R	gtttaactttaagaaggagatatacATGAAGCCGGAAGTTGAG
NEB_fas1_F	actttaagaaggagatataccATGGACGCTTACTCCACAAG
NEB_fas1_R	ctgtgatgatgatgatgatggctgctgccttagtggtgatgatggtgatgGGATTGTTCATACTTTTCCCAG
AT_S274A_F	GTGCTACAGGTCACGCCCAAGGTTTGGTTAC
AT_S274A_R	AACCACCACCGGCCAACTCAATGG
ER_H740A_F	GTGGTAGAGGTGGTGGTGCACATTCTTTCGAAGATG
ER_H740A_R	AGTTGTACAACAATGGCAAGCTGACGGG
DH_H1564A_F	GGTGATTTGAATCCAATTGCCGTTTCACGTCATTTTG
DH_H1564A_R	CCCATTTGCTGTCCACCAGTCATGC
MPT_S1808A_F	CTACTTTTGCTGGTCACGCGTTAGGTGAGTATGCTG
MPT_S1808A_R	CCCATTTGCTGTCCACCAGTCATGC
KR_Y839A_F	GACAACTTGGATTCTGACGCCATACCATCACCACCG
KR_Y839A_R	TCCATTCGCGGCCATTCCAGAACA
AT_S274_seq	ACCCTTCAAATACCCCAGACA
ER_H740_seq	TGTCCCATCATTGGAAGTTGC
DH_H1564_seq	TCACGGTAACCCTGTTGTTG
MPT_S1808_seq	GAGCACAGTACTTCTTACACA
KR_Y839_seq	CTTAAGAATGATGGGTTGTGTC

Supplementary Table 2. Data collection and image processing for cryoEM maps shown in figure 2 and supplementary figure 3.

Data Collection	WT Apo	WT + acetyl-CoA + malonyl-CoA + NADPH	KR(Y839A) + acetyl-CoA + malonyl-CoA + NADPH	AT(S274A)-MPT(S1808A) + malonyl-CoA	DH(H1564A) + acetyl-CoA + malonyl-CoA + NADPH	ER(H740A) + acetyl-CoA + malonyl-CoA + NADPH	AT(S274A)-MPT(S1808A) + acetyl-CoA
Microscope	FEI Tecnai F20	FEI Tecnai F20	FEI Tecnai F20	FEI Tecnai F20	FEI Tecnai F20	FEI Tecnai F20	FEI Tecnai F20
Camera	Gatan K2	Gatan K2	Gatan K2	Gatan K2	Gatan K2	Gatan K2	Gatan K2
Voltage	200 kV	200 kV	200 kV	200 kV	200 kV	200 kV	200 kV
Magnification	34,483x	34,483x	34,483x	34,483x	34,483x	34,483x	34,483x
Pixel size	1.45 Å	1.45 Å	1.45 Å	1.45 Å	1.45 Å	1.45 Å	1.45 Å
Exposure	36 electrons/Å^2	36 electrons/Å^2	36 electrons/Å^2	36 electrons/Å^2	36 electrons/Å^2	36 electrons/Å^2	36 electrons/Å^2
Exposure rate	1.25 Electron/Å^2/Sec	1.25 Electron/Å^2/Sec	1.25 Electron/Å^2/Sec	1.25 Electron/Å^2/Sec	1.25 Electron/Å^2/Sec	1.25 Electron/Å^2/Sec	1.25 Electron/Å^2/Sec
Number of Frames	30	30	30	30	30	30	30
Defocus Range	1.0-3.5 μm	1.0-3.5 μm	1.0-3.5 μm	1.0-3.5 μm	1.0-3.5 μm	1.0-3.5 μm	1.0-3.5 μm
**Image Processing**							
Frame motion correction	CryoSPARC V2	CryoSPARC V2	CryoSPARC V2	CryoSPARC V2	CryoSPARC V2	CryoSPARC V2	CryoSPARC V2
CTF estimation	CTFFIND4	CTFFIND4	CTFFIND4	CTFFIND4	CTFFIND4	CTFFIND4	CTFFIND4
CTF cutoff	5.0 Å	5.0 Å	5.0 Å	5.0 Å	5.0 Å	5.0 Å	5.0 Å
Particle selection software	cryoSPARC	cryoSPARC	cryoSPARC	cryoSPARC	cryoSPARC	cryoSPARC	cryoSPARC
Micrographs Used	47	38	161	43	38	36	47
Particle image motion correction	Alignpart_lmbfgs	Alignpart_lmbfgs	Alignpart_lmbfgs	Alignpart_lmbfgs	Alignpart_lmbfgs	Alignpart_lmbfgs	Alignpart_lmbfgs
Total particles picked	4,957	4,159	28,411	8,415	6,052	8,596	7,409
Particle images contributing to final map	3,487	2,664	19,804	5,073	3,590	3,771	5,739
Reconstruction Software	CryoSPARC V2	CryoSPARC V2	CryoSPARC V2	CryoSPARC V2	CryoSPARC V2	CryoSPARC V2	CryoSPARC V2
Symmetry Applied	D3	D3	D3	D3	D3	D3	D3
B-factor for sharpening	-122.4	-97.3	-125.7	-338.5	-203.7	-98.8	-81.3
Resolution (FSC = 0.143)	5.1 Å	5.0 Å	4.6 Å	6.6 Å	5.8 Å	4.7 Å	4.6 Å

Supplementary Table 3. Data collection and image processing for D3 refined maps shown in figure 3 and supplementary figure 9.

**Data Collection**	**DH(H1564A) + acetyl-CoA + malonyl-CoA + NADPH**	**ER(H740A) + acetyl-CoA + malonyl-CoA + NADPH**
Microscope	FEI Tecnai F20	FEI Tecnai F20
Camera	Gatan K2	Gatan K2
Voltage	200 kV	200 kV
Magnification	34,483x	34,483x
Pixel size	1.45 Å	1.45 Å
Exposure	36 electrons/$Å^2$	36 electrons/$Å^2$
Exposure rate	1.25 Electron/$Å^2$/Sec	1.25 Electron/$Å^2$/Sec
Number of Frames	30	30
Defocus Range	1.0-3.5 µm	1.0-3.5 µm
**Image Processing**		
Frame motion correction	MotionCor2	MotionCor2
CTF estimation	CTFFIND4	CTFFIND4
CTF cutoff	5.0 Å	5.0 Å
Particle selection software	Relion 3.0	Relion 3.0
Micrographs Used	329	258
Total particles picked	27,032	32,037
Particle images Contributing to final map	15,767	22,642
Reconstruction Software	RELION-3	RELION-3
Symmetry Applied	D3	D3
B-factor for sharpening	-215.3	-210.2
Resolution (FSC = 0.143)	4.4 Å	4.2 Å

Supplementary references:

1. Smith, J. M. XIMDISP—A Visualization Tool to Aid Structure Determination from Electron Microscope Images. *J. Struct. Biol.* **125**, 223–228 (1999).

2. Leibundgut, M., Jenni, S., Frick, C. & Ban, N. Structural basis for substrate delivery by acyl carrier protein in the yeast fatty acid synthase. *Science* **316**, 288–90 (2007).

3. Nguyen, C. *et al.* Trapping the dynamic acyl carrier protein in fatty acid biosynthesis. *Nature* **505**, 427–31 (2014).
