# Supplementary material for: Steric occlusion regulates proximal interactions of acyl carrier protein domain in fungal fatty acid synthase
Source: Commun Biol. 2020 May 29;3:274. doi: 10.1038/s42003-020-0997-y (PMC7260205; doi:10.1038/s42003-020-0997-y)
Supplement: Supplementary file 2 — Description of Additional Supplementary Files [file 42003_2020_997_MOESM2_ESM.pdf]

Description of Additional Supplementary Files

File Name: Supplementary Data 1
Description: Whole plasmid sequencing results for FAS1 and FAS2 genes clone from S. cerevisiae genomic DNA into bacterial expression vectors.

File Name: Supplementary Data 2
Description: Sequences of fungal species used in multi-sequence alignment. Sequences are presented in FASTA format.

File Name: Supplementary Data 3
Description: Source data for Figure 1d
